# Caring in the Context of Systems: Service Provider Perspectives on the Mental Health Needs of Newcomer Young Men

**DOI:** 10.1177/2752535X231217211

**Published:** 2023-11-22

**Authors:** Mia Tulli-Shah, Carla Hilario, Bukola Salami, Josephine Pui-Hing Wong

**Affiliations:** 1 University of Calgary, Calgary, AB, Canada; 2 UBC Okanagan, Kelowna, BC, Canada; 3 Toronto Metropolitan University, Toronto, ON, Canada

**Keywords:** mental health, young men, migration

## Abstract

In this study, we applied an intersectional framework to explore service providers’ perspectives on the mental health needs of newcomer young men. We conducted focus groups and interviews with 26 service providers in Edmonton, Calgary, and Vancouver, Canada. Findings show that service providers made sense of young men’s mental health needs and service access in the context of systems. We identified three interconnected themes: newcomer young men’s senses of self in relation to macro-systems, including racism and economic marginalization; settling well as a determinant of mental health; and systems capacities and interdependent resilience. While service providers are engaged in cross-sectoral work in support of newcomer young men’s mental health, this work is not being sufficiently supported. Further work is needed around cross-sector capacity bridging and advocacy, as well as the tailoring of services to young men without the assumption and reinforcement of gender stereotypes.

## Introduction

Mental health is gaining research and policy attention globally with the World Health Organization citing mental health as the most significant health issue currently facing young people.^1^ In Canada, suicide rates of men are about three times higher than those among women, and suicide is the second leading cause of death among young people aged 15 to 34.^2^ The mental health of newcomer youth has also received considerable research and policy focus globally and in Canada.^3,4^ There is evidence from the United States^5^ and Canada^6^ of links between experiences of discrimination and mental health challenges. However, relatively little focus has taken an intersectional analysis in paying attention to the mental health needs of newcomer young men specifically. Newcomer young men experience a unique set of circumstances and factors that shape their access to mental health services in Canada. These factors include social, economic, and political challenges pre-, during, and post-migration that can affect their mental health in profound ways.^7^ Such challenges can include experiences with violence or instability prior to or during migration,^8^ and uncertainty, financial strain, and encounters with discrimination upon arrival in Canada.^9,10^ Many of these post-migration challenges stem from anti-immigrant hostility and racism in Canada.^11^ Despite a reputation as welcoming to newcomers, Canada’s racist immigration legacy^12^ has extended into current anti-immigrant policies such as the routine detainment of irregular migrants (those who enter or remain in countries without authorization) and those seeking asylum,^13^ and credential deskilling.^14^ Further, anti-immigrant and xenophobia sentiment runs strong throughout much of the country.^10,15^

There has been some attention paid to newcomer young men’s mental health experiences,.^4,11,16^ Within the limited literature on newcomer young men’s mental health, a few Canadian studies have shown that newcomer parents perceive greater physical aggression from their sons than daughters.^17,18^ Evidence suggests direct links between child aggression and mental illness.^19^ At the same time, immigrant boys have reported higher rates of discrimination relative to girls, which in turn predicts lower levels of self-esteem and perceived social competence in peer relationships.^20^ Protective factors against emotional distress may also vary by gender. Research has shown that connectedness to culture may act as a protective factor against despair for young men, but less so for young women, while connectedness to schools was associated with lower odds of stress for young women but not for young men.^21^ These gendered differences suggest the need for targeted approaches to fostering the mental health and wellbeing of newcomer young men.^11^ Despite this knowledge, there continues to be limited research into how gender, age, and migration and resettlement shape newcomer young men’s mental health needs with accessing services, a focus of this study.

Research on the perspectives of service providers working with newcomer young men is also scant. Some studies have explored service providers’ views on barriers to mental health care access that newcomers face. For example, settlement service providers in Ontario have described barriers to mental health service access faced by newcomers, including cultural insensitivity and lack of resources.^22^ Findings from Alberta indicate language barriers and cultural understandings of mental health as barriers to access.^23^ In both studies, stigma was identified as a barrier to care.^22,23^

Research about newcomer young men’s mental health has largely focused on the role of cultural sensitivity,^24,25^ often ignoring structural and relational analysis.^7^ To fill gaps in our understandings of how migration, gender, age and intersecting axes of structural determinants shape newcomer young men’s mental health and access to services, this study sought to understand the structural and systemic contexts that shape mental health service provision for newcomer young men. In this exploratory study, newcomers broadly refer to individuals who moved to Canada within the last 10 years. Existing literature, albeit limited, shows that the mental health of immigrant youth is shaped by multiple determinants, such as racial discrimination, changing family dynamics, mental health literacy, access to integration, identity development, and sense of belong.^26,27^ Therefore, we intentionally engaged service providers beyond the health sector, including community youth workers, settlement service providers and nurses, who work with immigrant and refugee young men aged 25 and under. Our research questions were: What are the mental health needs of newcomer young men? How are service providers meeting these needs?

## Theoretical Lens

We drew on the theoretical lens of intersectionality in this study,^28^ which postulates that individual experiences are shaped by overlapping social identities, including gender, race, class, immigration status, etc. and by intersecting systems of privilege and oppression that work to advantage or marginalize different groups in multiple ways.^28^ In using this lens, we answer^29^ call to centre structure, rather than behaviour or culture, in understanding immigration-and-settlement as a social determinant of health. By centering structure, we explore individual experiences within the context of social systems that shape health and access to health services in the Canadian context.

## Methods

Data collection was completed in three research sites across western Canada: the metropolitan areas of Edmonton and Calgary in Alberta, and Vancouver in British Columbia. The study sites were organizations and programs that provided services to newcomer young men, including - but not limited to - mental health services. Some of these immigrant youth programs were housed under broader organizations such as school boards. Services provided by these organizations included settlement services, legal information, employment services, English classes, interpretation and translation, child/youth/family support, violence prevention, and health and counseling. The focus groups and interviews were conducted in-person on site or at a location in the field that was convenient for the participant. The areas of Edmonton, Calgary, and Vancouver were chosen because they have the highest proportion of newcomers in Western Canada. In 2016, Alberta received 16.6% of permanent residents to Canada with most settling in Calgary and Edmonton, while BC received 12.8% with the majority settling in Vancouver.^30^ The research team had existing networks in the three sites, which facilitated collaboration, recruitment, data collection, and knowledge exchange. The study received ethical approval from University of Alberta and Ryerson University research ethics boards.

## Recruitment, Data Collection, and Analysis

Recruitment and data collection was completed in 2019. Participants were recruited from newcomer-serving agencies and related organizations in the three sites using purposive and snowball sampling strategies. Two focus groups were conducted in Edmonton, two focus groups and two interviews in Calgary, and six interviews in Vancouver. To enhance access of participation, individual interviews were conducted for those who wished to participate in the study but could not join focus groups due to time constraints or a preference for greater anonymity. In total, 26 participants took part in the study.

Interviews and focus groups were conducted by two research team members (Tulli, Mia and Hilario, Carla). Focus groups and interviews were guided by open-ended questions, for example, ‘what are the key mental health challenges faced by immigrant and refugee young men at your organizations?’ The focus groups were audio-recorded and transcribed verbatim by a professional transcriptionist before being uploaded into NVivo qualitative software. Participants provided informed written consent to this recording and their participation in the study. Our data analysis was guided by our key objective to gain knowledge on: (1) the mental health needs of newcomer young men; and (2) how service providers and the healthcare system meet these needs. We applied thematic analysis^31^ through reiterative processes, whereby team members (Tulli, Mia and Hilario, Carla) familiarized themselves with the transcripts before creating a preliminary coding tree. These codes were then reviewed and refined as further data was coded and themes and patterns emerged. Researchers then worked together to define themes and consider how themes related to one another. Any differences in coding that did arise was discussed between team members before consensus was agreed to. To maintain anonymity, providers chose pseudonyms to use during focus groups and interviews. These pseudonyms are attributed to quotes presented in our findings.

## Results

### Participant Demographics

Of the 26 participants, the majority (*n* = 20) worked for settlement and newcomer agencies. Three worked for organizations that delivered both health and settlement services, two worked in health services, and one worked for a mental health organization. More than half identified as men (*n* = 14). Approximately one third of participants reported that at least 50% of their clients experienced mental health issues in the last 6 months. A total of 15 participants self-identified as an immigrant or refugee themselves. Information on participants’ racial/ethnic identity can be found in Table 1: Participant demographics. Although this paper is concerned with the experiences of newcomer young men, we include demographic data about service provider participants as part of our overarching intersectional analysis. Provider positionalities likely shape their perspectives in relation to their own experiences in providing services, as well as their understandings of the experiences of those they provide care to.

In this study, participants expressed their perspectives on the mental health needs and access to care among newcomer young men in the context of systems. Detailed analysis of participant narratives resulted in three key themes in relations to systems. Our first theme, **a sense of self in relation to systems**, included connections between mental wellbeing and the systems of migration, racism, and economic marginalization. The second theme that arose, **settling well as a determinant of mental health**, included service provision in connection to coordination across systems and advocacy. The third theme, **system capacities and resilience**, included issues related to lack of funding and wait times for service access, limitations of mainstream mental health care providers, mental health stigma, and opportunities to promote resilience among clients.

### Theme 1: Sense of Self in Relation to Systems

Most service providers in this study discussed the mental health needs of newcomer young men through the language of systems and layering, i.e., cumulative and interlocking layers of access barriers. They explained how their clients’ senses of self and individual experiences are formed and experienced in relation to a multiplicity of systems. These systems could be described as operating at the micro-level as personal identities, social memberships, and interpersonal relationships, and are shaped by macro-level systemic racism, economic marginalization, and other systems of exclusion which act as social determinants of health. The mental health and wellbeing of newcomer young men was perceived to be shaped by their identity and social positioning, migration and settlement experiences, and personal and relational resources, all of which layered on one another.

I think we share such compelling layers of factors, eh? There’s the pre-migration experience. There’s the post-migration reality. There’s the internal family dynamics. There’s the community dynamics. And then at the core, the person’s own sense of who he is, and the relationship with systems hey? And they all interplay. Some are contributing factors. Some are, you know, multiplying factors, right? So, it’s a very complex layer of influences, isn’t it? Yeah. Yeah. And much of it, of the context, is quite unique, I guess, to refugee or immigrant youth [Mei Mei, Edmonton].

Mei Mei explicitly described mental health needs of newcomer young men as shaped by layering internal and external dynamics. As they explain, the result is a unique set of mental health needs that are unique to the experience of being an immigrant or refugee youth.

Within these intersecting systems, the sense of self for newcomer young men is often shaped by their experiences of racism and discrimination. These experiences may also discourage access to services if young men feel stigmatized within and outside of the Canadian health care system. Witnessing discrimination against their families can also impact young men. As Harrison explains, his son noticed him being racially profiled and knew this was unfair at a young age.

I have been living in Canada for 23 years, and my son used to tell me, say, you don’t notice how they treat you dad?…They treat you very bad sometimes no? Because your English is not good or whatever, he said, but they treat you bad, and my, my son, he was 7, so after 5 years he was completely bilingual, and he used to tell me those kind of things, no they were not very nice with you. And this, and then after that I started to notice when they were not very good with me, no? But now I know what to say…I know that I can say, so what’s happening, what’s the problem, or what are you, like I can go to a store, and the police, one of the guard is following me, no?…Now so, why are you profiling me? Because my skin colour, or because my wardrobe? So, what’s the problem? [Harrison, Vancouver].

Harrison recalled experiences of being profiled based on his racialized appearance and language skills. His son’s awareness to this discrimination shaped how he also saw himself in Canada, as well as his sense of self. He discussed further:

Because, you don’t have a job, you don’t speak the language…we have many problems because, yeah, sometimes you feel like not very welcome, as well, no? There are things about the colour of your skin, or your accent or whatever, that they, make things very difficult for you [Harrison, Vancouver].

Here, he explains how many of his clients faced experiences of racism and discrimination which caused stress and shaped the ways they saw themselves in Canada, impacting their emotional wellbeing and mental health.

Alongside, and often interwoven through systemic racism and discrimination in Canada, systems of economic marginalization were perceived as affecting newcomer and racialized families. Factors such as unaffordable housing, lack of settlement and social services supports, and systemic deskilling of newcomers’ professional credentials were seen to shape newcomer young men’s understandings of themselves and their potential while also preventing their access to meeting their basic needs for mental health and wellbeing. Providers in Vancouver expressed particular concern about the lack of affordable housing and its effects on the lives of newcomer youth and their families. One participant in Edmonton highlighted unique circumstances for some newcomers, particularly refugees:

So, it’s all kind of all at once, and at the same time, they’re worried about their families, their younger siblings. Maybe the money they’re getting from the government to support them [refugees] for that first year or second year isn’t enough, so that they have to work and provide for their families as well. Like I mentioned earlier, there’s always that burden on them [young men] to support their family, because that’s what they’ve been doing for most of their lives [Hala, Edmonton].

As Hala explains, economic marginalization may be experienced in different ways by refugee youth, especially young men, who may have taken economic responsibility for their families during their migration journeys to Canada.

Finally, a sense of self was also seen to be shaped by experiences of trauma. The participants spoke about the ways in which trauma can affect how people see themselves and their abilities to access supports and services. As one participant described,

Particularly when I look at boys from Syria, the Sudan, and Colombia, Afghanistan. Look what they saw. What do you think they’re seeing? What do you think they’re experiencing? Why don’t we look at PTSD? Why don’t we look at attachment, particularly those boys who’ve seen their fathers killed in front of them, or their mothers killed in front of them? Maybe it’s safer not to attach to anything [Jess, Calgary].

Here Jess explains that PTSD may make it difficult for people to form attachments or bonds with care providers, an issue for providers attempting to build trust and relationships with their clients.

### Theme 2: Settling Well as a Determinant of Mental Health

Providers identified “settling well” as an important determinant of mental health among newcomer young men. Good settlement requires coordination between different systems (including health, settlement, social, education) to meet the needs of newcomers. They also discussed the importance of being attentive to interactions between policy and provider sectors and understanding service access from a systems-oriented, intersectional perspective. They reported that that many newcomer young men had a lot going on in their lives and often faced multiple, intersecting, and layered sources of stress. Many of these challenges were related to settlement and integration, including, experiences of racism and discrimination, trauma from migration experiences, and economic marginalization and financial stress. Therefore, providers emphasized the importance of ‘settling well’ as integral to newcomer young men’s mental health and wellbeing. As one participant explained, access to settlement and social services facilitate access to employment, housing, language classes, and community engagement are important alongside mental health care.

When people settle well … it helps their mental health, right? And when they integrate well, it helps their mental health. So any of the settlement programming… Like when you’re helping someone with employment, that ultimately helps with their mental health… Language, development, all of these things. But whether [mental health] is on the forefront, I think that’s more of an emerging issue within the agency [Monica, Calgary].

This theme of settling well largely referred to coordination between systems and advocacy for changes in practice, with implications for mental health promotion.

In order to support newcomers in settling well, participants highlighted systems-level and comprehensive solutions to meeting these needs. For example, access to settlement services, social supports and safe spaces were discussed alongside access to mental health care for newcomer young men. Cross-sector/cross-system efforts were seen as paramount to realizing this coordination. As Monica described above, resettlement supports offered necessary protections against systems of economic marginalization. However, these important social determinants of mental health may be missed by providers and policy makers who view mental health care through a singular health sector lens.

[Newcomers’] psychological experiences might manifest in physical symptoms. But when a person comes in and says, “I have chest pain,” it’s not the instinct of the physician to say, “Well, when did you come to Canada? Are you having access to… are you able to access? Like do you have a job? Do you have access to food? Do you have access to shelter?” These are not common questions that we ask, so it’s only after hours of being in the Emergency Department and having all these like physical tests or objective tests done, more conversation will lead to [discussion of settlement challenges] [Elisa, Vancouver].

Elisa, a nurse, explains that isolating crisis responses to a purely health care lens, can mean providers may miss social determinants of health, especially those related to migration. This, in turn, may prevent holistic and effective service provision.

Cross-sector advocacy was viewed a key part of service provision that supports the mental health needs of newcomer young men. Participants across research sites identified education and social inclusion, vis a vis belonging in schools, as important determinants of good mental health. They expressed concerns that newcomer young men are not set up well for this success. As one participant recounted,

I had one youth who was getting in trouble at school because he couldn’t sit in a classroom.… he had to leave. He had to get up and walk around, whether it’s around the school or, you know, he would make excuses that he’s going to the bathroom or going to his locker. But he had to get up at every class he had. So at one point he started just not going to school at all. And I actually talked to him and he said, “They’re going to kick me out of school because of… because I’m not attending.” And I just had the conversation with him, “Why aren’t you attending?” He’s like, “Every time I sit in that classroom I feel like the walls are closing in on me.” So he said that he had to just get up and walk around, just so he can breathe again [Hala, Edmonton].

In this case, the student was getting in trouble because the school viewed his behaviour as problematic, failing to recognize that the student left class as a response to trauma. A narrow or singular lens may miss mental health issues related to trauma, economic marginalization and/or discrimination*.* Providers suggested that those working within education systems who do not think or work in cross-sectoral ways may miss seeing the whole picture when it comes to their students. In this example, the settlement worker was able to identify a need that was missed and advocate for more a responsive approach to meet the youth’s needs.

### Theme 3: System Capacities and Interdependent Resilience

The theme of system capacities and interdependent resilience refers to the challenges providers face in supporting their newcomer clients’ mental health and wellness. Limited system capacity, i.e., lack of funding and long wait times for service access, limitations of mainstream mental health care providers, and mental illness stigma leave many newcomer young men without sufficient support. Providers explained that capacity limitations in one system or sector reverberate across others and the failure to understand these system connections and the intersectional challenges faced by many newcomer young men means that success in one sector may be ineffective if supports are lacking in others. Despite these barriers and system limitations, providers observed that their clients often aided in their own care through individual and collective resilience. They explained the importance of tapping into this interdependent resilience, personal strengths and relational resources to develop strength-centered programming.

For example, providers described how their clients often experienced long wait times when attempting to access mental health services. They explained how services also needed to extend beyond medical care and that settlement and social services were also part of mental health care. Providers emphasized that settlement challenges related to housing and economic stability may be felt especially acutely by young men who arrived in Canada as refugees because many took on economic responsibility for their families during migration journeys. They also explained how a lack of funding for settlement services and targeted supports can leave newcomer young men behind, especially when funding mechanisms may leave some newcomer-serving organizations with low capacity and long waitlists. As one participant described,

It’s a big challenge when you – and excuse my language – when you have like a mainstream agency that has like all this funding and it’s big… like they’re hiring therapists… that don’t understand the culture, or the needs, and it’s just sitting there. Do you know what I mean? Meanwhile, we have 200 people on our waiting list. That’s just… it’s not fair, and it doesn’t make sense to us or like to our clients [Hala, Edmonton].

This provider referred to challenges related to funding mainstream mental health care provision while newcomer-serving organizations require additional funding to provide needed mental health services to their clients.

Many participants felt that mainstream providers are ill-equipped to support the mental health needs of newcomer young men because they often did not understand their needs within the context of systems, including layered and intersecting social identities and issues related to migration and settlement. Cross-sectoral partnerships and efforts to bridge capacity were not always practiced by mainstream mental health service providers. Providers called for mental health service providers to move beyond a biomedical system of intervention, and the reliance on prescribing medication, to meet the intersecting and layered factors shaping individual experiences with mental health. As Mei Mei expressed,

And we wish other people could understand these layers, hey? Most of the time, others who offer help, they don’t see the layers, and so they’re treating what they think they know, hey? [Mei Mei, Edmonton].

Here Mei Mei expresses frustration with mainstream approaches to mental health care that do not consider mental health within the context of layered factors, especially those factors which may be unique to newcomer communities, such as pre-migration trauma, settlement stresses, and racism and discrimination.

Access to care was also linked to stigma related to mental illness and assumptions about masculinity. To address this, providers described the use of strategies to help establish trust and to encourage their clients to seek supports, including home visits, somatic and action-based activities, and using language of wellbeing rather than mental health. They explained that stigma was an important factor preventing many newcomer young men from accessing mental health supports.

It’s like the shame for us, we have this person in the house, or the stigma. And most of them, they don’t…go to the counselor or therapy because they feel…ok. This stigma’s still also alive [Jwan, Edmonton].

Jwan explained that it was rare for their clients to proactively seek out mental health services and supports. Instead, it was often their families who saw them struggling and encouraged them to access counseling or other services.

Participants reported their clients’ resilience was a key resource providers can draw on in supporting them. Their clients’ personal strengths, such as being resilient, creative, curious, determined, and having a sense of humour, helped organizations provide appropriate support. In several cases, providers explained that the process of migrating itself requires resilience. While coming to a new country, a new system, often creates challenges, such as social isolation, income instability, and encounters with discrimination, providers noted the need to acknowledge the resilience of newcomer youth and their families.

Strength-based programming could build on and support the development of clients’ senses of self and belonging, as well as supporting young men in building relationships with others. These relationships were seen to function as motivation to seek support for one’s own wellbeing. Some providers defined resilience in the context of relationships and a sense of responsibility to others.

Family [lalong lalo na/first and foremost] I feel is very, very important [sa kanya yon/to him]. And how they eventually trusted him, and how they made him feel like a [kuya/older brother]. That was very helpful. I felt almost a sense of responsibility that I have to look after others. I feel that that helped him a lot. Because prior to that there was no sense of responsibility. He was only looking after himself. He was only… it was just himself. But when they gave him more responsibility, “You have to look after your younger siblings. You have to be a good role model to them,” I feel that it made him realize, “Okay, I have to make something out of myself.” It helped a lot for his sense of responsibility [Francine, Vancouver].

As Francine discusses, supporting her client meant developing his sense of responsibility. She was able to support his wellbeing by drawing on his already existing relationships and desires to be well for his siblings.

## Discussion

In this study we explored service provider perspectives on the mental health needs of newcomer young men as well as the opportunities and challenges they face in addressing those needs. Our findings indicate that providers serving this population understand their clients’ needs in intersectional ways and are attentive to the multiplicity of systems these youth are connected to. This is consistent with research by^32^ that identified the importance of newcomer youth’s microsystems (i.e., school and family) to their educational success. Our study adds to this analysis by considering microsystems and individual experiences of trauma in relation to broader systems of racism and economic marginalization.

The participants in our study discussed ways that trauma can make daily life difficult for their clients. They explained how those who suffer from PTSD related to pre-, during, and post-migration, such as exposure to violent conflicts are often left unsupported, especially by schools in Canada. Participants understood that experiences of trauma are associated with psychological barriers that make participation in social activities difficult, leading to increased isolation and loneliness. They may also create difficulties in schools, where disruptive behaviours are not recognized as trauma responses and coping skills.^33,34^ The mental health recovery of newcomer young men within Canadian systems is also associated with how trauma was felt and addressed. Systemic discrimination, such as racism and economic marginalization, often reinforces a sense of insecurity or feeling unsafe, which may exacerbate existing mental health challenges, such as PTSD and depression, among immigrant young men.^29^

These findings confirm existing evidence that health care providers who lack training in transcultural and trauma-informed care often have a difficult time recognizing complex trauma in mental health assessments.^35^ In more recent times, issues related to this may have been exacerbated by the COVID-19 pandemic because service provision was moved largely to remote online delivery.^36^ Further research may be needed to investigate the effects of remote service provision on transcultural and trauma-informed mental health assessments.

Our study also showed that mental health and wellbeing itself is often shaped by one’s position in relation to systems of violence, echoing findings from the United States that showed perceived discrimination has an effect on the physical and mental health of refugees.^37^ Evidence of discrimination as a health determinant is also available in Canada.^38^ Experiences of racial profiling, as reported in this study, shape how newcomers, including service providers, understand their place in Canadian society.

Considerable research has found that stigma surrounding poor mental health and mental illness can impact mental health and wellbeing and access to supports.^22,23^ Our findings confirm this data. However, participants in our study also explained their work in breaking down mental health stigma through relationship and trust-building, signalling the need for continued efforts toward breaking down mental health stigma newcomer communities. Another issue identified in our study is whether clients want to see providers from their own community. Some clients were more willing to discuss challenges with providers from similar cultural backgrounds because they better understood their lives, while others were only willing to seek care from providers outside of their cultural communities for greater privacy.

In addition, we wanted to consider ways that mental health stigma may be a systems capacity limitation, rather than the responsibility of specific cultural and ethnic communities. Other recent work around newcomers and mental health stigma in Canada has also begun conceptualizing stigma as a failure of mainstream mental health care system. For example,^39^ found that existing strategies to reduce mental health stigma have largely ignored cultural and gendered contexts of stigma and failed to engage leaders within cultural and ethnic communities as mental health advocates. Again, this affirms a need for cross-sector and cross-system coordination in supporting newcomer young men’s’ mental health and wellbeing and pursing health equity more broadly.

We found that providers are attentive to the ways gender norms may vary across demographic groups. This echoes prior work showing how hegemonic gender scripts across many cultural contexts may disallow possibilities for vulnerability in different ways, often shaped by other intersecting axes of identity.^4^ For example, age may play a role in young men’s willingness to engage in mental health services and programming. One Canadian study found that younger Asian men were more resistant to stigma around mental health than older Asian men, independent of age at time of migration, indicating that age may influence the degree to which newcomer men are able to resist or overcome mental health stigma and to seek help.^40^

Recently, criticism has been levied against Canada for failure to meet international calls to implement Health in All Policies (HiAP) approaches to care provision and sector coordination.^41^ Such an approach centres the pursuit of health equity by promoting intersectoral policymaking and cross-sectoral collaboration.^41,42^ Some countries have even specifically focused HiAP cross-sectoral approaches to mental health care in what has been called Mental Health in All Policies (MHiAP).^43^ MHiAP approaches have gained increased traction in policy discussions in North America, prompted by COVID-19 pandemic simultaneous interruptions to service access social connections.^44^ Recognizing the need for a HiAP approach to health equity, researchers have also began calling for an extension to a Migration and Health in All Policies approach.^45,46^ Despite being a member of the World Health Assembly (which committed member states to improve health equity through a HiAP approach in 2015), Canada has not coordinated action across sectors in meaningful ways.^41^ Our findings show providers are calling for greater coordination across systems and policy sectors. These results are congruent with reports from settlement service providers in Newfoundland and Labrador who expressed a similar need for greater collaboration between schools, community organizations, various service agencies and the business community in supporting newcomer youth.^32^

Even in cases where governments are not utilizing HiAP or MHiaP approaches, civil society is being increasingly recognized for its role in addressing social determinants of health.^47^ A recent study from Finland shows intersectoral collaboration around mental health promotion offers “collaborative advantage” including capacity building in professional practice and resource sharing.^48^ Our study shows this work is being done in Canada as well. However, despite evidence that many newcomer young men have unique mental health needs and face unique barriers to access of care, there has been a lack of government or mainstream service provider response. The work being done by providers serving newcomer young men is under-supported by government and mainstream providers across sectors. System reform, including responsive funding of this work and the incorporation of intersectional approaches in mainstream mental health care are necessary.

In emphasizing the need for capacity bridging across service sectors to support the needs of newcomer young men, providers especially focused on the importance of partnerships and advocacy work in school settings. During settlement, integration in Canada may take place across different settings. Schools are an important area to integrate newcomers into Canada, as they can create a ‘safe space’ for integration and contribute to social and emotional well-being.^49^ Schools are also important facilitators of teaching the official languages of the receiving country. Fluency in English was linked to a reduced likelihood of depressive symptoms.^50^ Our findings also confirm evidence on education policy from the United States that shows tailored approaches to supporting newcomer secondary school students is important for academic success and mental wellbeing.^51^

Studies consistently show inequitable access to health care services in Canada, with newcomers facing unique barriers to access and often times long waitlists for services.^52^ Yet, few studies have explored the inter-relatedness of health, settlement, and social service access. Participants in our study explained the importance of coordination between these sectors to ensure people settle well and receive effective mental health supports quickly. This may be an area for further work. The study findings suggest that shortcomings in providing settlement and social support may also render mental health care ineffective because such care will miss a key factor in these young men’s mental wellness. In addition, providers explained how even those young men who are able to access mental health services without long wait lists may not receive appropriate care if they are still waiting for language training, affordable housing, income supports, and so forth.

Mainstream mental health service provision is based largely in a biomedical system of care. Care oftentimes fails to consider the ways that personal experiences are connected to systemic issues.^53^ Participants in this study explained why this model fails many newcomer young men: their mental health is often shaped by compounding layers of identities, experiences, and personal and relational resources. For those in need of mental health care, economic marginalization, stigma, and racism and discrimination often act as key barriers to access of care.^9,54^

Effective program planning focuses on fostering newcomer young men’s personal strengths and bolstering their relational resources through an emphasis on trust-building and connection. Strengths-based program planning involves the recognition and enhancement of strengths, such as strong family ties and collective resilience. This offers a unique perspective on resilience as a significant body of literature has focused on how programming can help bolster resiliency in newcomer youth,^55,56^ with limited attention on interdependent individual and collective resilience in newcomer communities.

In our study, participants themselves overwhelmingly discussed intersectional issues around newcomer young men’s mental health and wellbeing and we saw our theoretical lens of intersectionality reflected back through provider understandings of their clients’ experiences. Often using the metaphor/descriptor of ‘layers’, almost all participants spoke about factors in intersecting, inter-related, and layered ways that can shape mental health for newcomer young men. This reflects how intersecting, inter-related, and layered relationships to systems interact with and compound on one another to shape individual identities and experiences.

Many participants described providing care in the context of systems, and several organizations were pursuing systems-level solutions by bridging capacity between their institutions and across sectors. It is important to note that we conducted our analysis through this lens and were thus acutely attentive to participant responses which reflected intersectional understandings of mental health. We make this explicit to account for ways our analytic biases may have shaped our analysis of data.

Despite this project’s focus on young men and on intersectionality, however, our findings do not offer nuanced accounts of how gender plays a role in mental health and mental health care needs of newcomer young men. The participants did not often attend to gender in describing the ways that they provided care to their clients in the context of systems. Instead, providers emphasized their clients’ senses of self in relation to other systems, like systems of race, migration, and economic security. While gender considerations were brought up in some conversations about program planning and cross-sectoral capacity bridging, other factors, such as trauma and economic concerns were often the focus in discussions with service providers.

Across the three research sites, considerations of gender did not feature heavily in participant responses, despite this study concerning young men specifically. We found that many providers did not identify gender as a key social determinant of mental health or access to services even when being asked explicitly about gender. When issues related to gender were discussed, these references largely focused on prevailing notions that young men are not as willing to talk about problems as young women or that they fear being seen as weak or vulnerable if they seek help, and how this has prompted program planning for newcomer young men to incorporate somatic-based programming for example. While creative approaches to programming may be helpful, there may be an overreliance on gendered assumptions either that young men are unable/unwilling to communicate, or that young women would not also benefit from somatic activities in mental health and wellness promotion. Overall, we found that gender was not a focus of program planning or in consideration around the mental health needs of newcomer young men. In a similar way, there was little discussion of age. This may point to a need for greater interrogation of these factors in future research.

## Implications

Despite research and policy evidence suggesting cross-sectoral approaches to health care are necessary for health equity, this approach has not been met with government support or system designs that further this work. While providers are employing HiAP practices, the Canadian health and social care systems are not providing sufficient support in this work. Our study also raises two implications for research and service provision. First, additional research is needed to examine how service providers can further bridge capacity with partner organizations and sectors to collectively deliver more responsive programming. Second, further work is needed to understand how capacity bridging can help mainstream mental health service providers tailor care for newcomer young men and at the same time account for nuance and differences within groups to ensure they do not assume or reinforce biases or stereotypes related to gender.

## Limitations

We collected data from immigrant service providers on their perspective of the experience of newcomer young men. A limitation of the study is that we did not collect data from young men themselves. We encourage future researchers to triangulate their data sources by collecting data from newcomer young men, service providers and other stakeholders. In addition, as discussed above, we found service providers reflecting our theoretical lens back to us through their conceptualizations of mental health and mental health needs in intersectional ways. It is important to recognize how researchers may be primed to identify aspects related to a theoretical lens, such as intersectionality, more readily if it guided the approach from the outset. Additionally, only one participant worked for a mental health organization. Finally, as we have engaged only 26 service providers, we recognize that their perspectives are partial and do not present the experiences of all newcomer young men.^57–63^

## Conclusion

This study explored the perspectives of service providers who support newcomer young men. We found that providers overwhelmingly understand their clients’ needs in intersectional ways and as shaped in relation to multiple systems. These systems include their interpersonal relationships and connections, their social positioning and identities as connected to systemic processes of racism and economics which disproportionately marginalize newcomers in Canada, and their experiences accessing resources across policy sectors.

In an effort to support the mental health and wellbeing of newcomer young men in their programs, providers delivered programs that centre their individual and collective resilience and that work to bridge capacity across health, settlement, social, and educational sectors. These efforts were seen to require additional government support. Our findings also suggest the need for nuanced considerations of gender in service provision to newcomer young men as well as in future research on youth mental health and wellbeing.

## Appendix

**Table 1.** Participant Demographics.

Racial/Ethnic Identity	*n*	Approximate Percent
South Asian	5	19
West Asian	4	15
East Asian	3	11
Southeast Asian	1	4
Black/African	3	11
Black/Caribbean	2	7.5
White	2	7.5
Latin American/Hispanic	1	4
Multiracial	1	4
No response	4	15

**Table 2.** Themes, Examples, and Implications for Policy.

Themes	Examples	Implications for Policy
Sense of self in relation to systems	Layering internal and external dynamics including racism and discrimination, economic marginalization, and trauma	Mental wellbeing was perceived to be shaped by interactions of identity and social positioning, including migration and settlement experiences, and personal and relational resources, all of which layered on one another
Settling well as a determinant of mental health	Access to settlement services, social supports and safe spaces were discussed alongside access to mental health care for newcomer young men	There is a need for cross sector comprehensive supports that connect settlement, educational, economic, and health care, as well as advocacy that extends across service sectors
System capacities and interdependent resilience	System capacity issues include lack of funding and long wait times, limitations of mainstream mental health providers, and impacts of mental illness stigma. Client resilience and interconnectedness can mitigate some of these barriers	To address issues related to systems-capacities, cross-sectoral and strength-based programming can build on client resilience, personal strengths, and relational resources
